# Dichloromethane Degradation Pathway from Unsequenced *Hyphomicrobium* sp. MC8b Rapidly Explored by Pan-Proteomics

**DOI:** 10.3390/microorganisms8121876

**Published:** 2020-11-27

**Authors:** Karim Hayoun, Emilie Geersens, Cédric C. Laczny, Rashi Halder, Carmen Lázaro Sánchez, Abhijit Manna, Françoise Bringel, Michaël Ryckelynck, Paul Wilmes, Emilie E. L. Muller, Béatrice Alpha-Bazin, Jean Armengaud, Stéphane Vuilleumier

**Affiliations:** 1 Laboratoire Innovations Technologiques pour la Détection et le Diagnostic (Li2D), Service de Pharmacologie et Immunoanalyse (SPI), CEA, INRA, F-30207 Bagnols-sur-Cèze, France; Karim.HAYOUN@cea.fr (K.H.); beatrice.alpha-bazin@cea.fr (B.A.-B.); 2 Génétique Moléculaire, Génomique, Microbiologie, UMR 7156 CNRS, Université de Strasbourg, F-67000 Strasbourg, France; emilie.geersens@etu.unistra.fr (E.G.); carmen.lazaro-sanchez@etu.unistra.fr (C.L.S.); amanna@unistra.fr (A.M.); francoise.bringel@unistra.fr (F.B.); emilie.muller@unistra.fr (E.E.L.M.); 3 Institut de Biologie Moléculaire et Cellulaire, UPR 9002 CNRS, Université de Strasbourg, F-67084 Strasbourg CEDEX, France; m.ryckelynck@unistra.fr; 4 Luxembourg Centre for Systems Biomedicine, University of Luxembourg, L-4362 Esch-sur-Alzette, Luxembourg; cedric.laczny@uni.lu (C.C.L.); rashi.halder@uni.lu (R.H.); paul.wilmes@uni.lu (P.W.)

**Keywords:** pan-proteomics, differential proteomics, *Hyphomicrobium*, dichloromethane, dehalogenation, *dcm* genes, genome sequencing, Nanopore

## Abstract

Several bacteria are able to degrade the major industrial solvent dichloromethane (DCM) by using the conserved dehalogenase DcmA, the only system for DCM degradation characterised at the sequence level so far. Using differential proteomics, we rapidly identified key determinants of DCM degradation for *Hyphomicrobium* sp. MC8b, an unsequenced facultative methylotrophic DCM-degrading strain. For this, we designed a pan-proteomics database comprising the annotated genome sequences of 13 distinct *Hyphomicrobium* strains. Compared to growth with methanol, growth with DCM induces drastic changes in the proteome of strain MC8b. Dichloromethane dehalogenase DcmA was detected by differential pan-proteomics, but only with poor sequence coverage, suggesting atypical characteristics of the DCM dehalogenation system in this strain. More peptides were assigned to DcmA by error-tolerant search, warranting subsequent sequencing of the genome of strain MC8b, which revealed a highly divergent set of *dcm* genes in this strain. This suggests that the *dcm* enzymatic system is less strongly conserved than previously believed, and that substantial molecular evolution of *dcm* genes has occurred beyond their horizontal transfer in the bacterial domain. Our study showed the power of pan-proteomics for quick characterization of new strains belonging to branches of the Tree of Life that are densely genome-sequenced.

## 1. Introduction

Continuing advances in high-throughput sequencing technologies are revealing increasingly large diversity in the microbial world [1]. Beyond its fundamental interest, this unsuspected diversity holds great promise for the discovery of novel enzymes for application in biocatalysis and bioremediation [2]. However, because a large part of DNA sequences retrieved from our environment is of unknown function, information derived from sequence only provides preferential access to enzyme classes that are already known, rather than to the much larger diversity of enzymes performing a given function that likely exist in nature.

Thus, complementary approaches to DNA sequencing are needed to rapidly identify new ways of performing a function of interest. Proteomics is one of the high-throughput omics techniques available today to gain information. In particular, comparative proteomics under different conditions of interest for the investigated function has the potential to detect differentially synthesised proteins, providing hints to their functional association [3]. The proteome of closely-related bacterial strains can be compared unifying all the genome sequences into a single protein database for interpreting shotgun proteomic data [4]. This pan-proteomics concept allows better quantitative proteome measurements and was recently applied to characterize the proteome of *Lactococcus lactis* strains [5], *Streptococcus agalactiae* strains [6], and mid-exponentially grown *Brucella* representatives [7], thereby promoting information attributions about unified groups of proteins within a given branch of the Tree of Life. The concept can be further extended to still uncharacterised isolates.

One microbial function that has attracted much interest is dehalogenation, also because of the wide range of potential uses for dehalogenase enzymes [8,9]. Many halogenated compounds are produced and present naturally on earth [10]. Industrial production of some of these compounds has led to the expansion and further evolution of specialized bacteria able to degrade such compounds and to use them as nutrients for growth. A number of dehalogenases have been discovered and characterised at the gene, protein, and enzymatic levels. Nevertheless, known enzymes with this function belong to a relatively small number of protein families, and may represent only a fraction of the diversity of the extant dehalogenase repertoire on our planet.

Cultivable strains that grow with a given halogenated compound, but in which the corresponding dehalogenase gene cannot be detected, hold great interest with respect to the discovery of novel dehalogenases. Dehalogenation of dichloromethane (DCM) represents a long-standing and thoroughly investigated paradigm in the field [11]. DCM is a naturally produced volatile and water-soluble toxic compound, and one of the major halogenated solvents intensively used by the industry. Thus, bacteria capable of degrading dichloromethane were among the first dehalogenating organisms to be discovered as part of initial efforts to address the issue of environmental contamination by organohalogens. Following the characterisation of DCM dehalogenases and gene identification, it was established that the DCM dehalogenase *dcmA* gene was very strongly conserved in all investigated aerobic DCM-degrading bacteria. To date, DCM dehalogenase is still the only growth-supporting dehalogenase acting on DCM that has been characterized at the molecular level [11].

The DCM-degrading strain MC8b used in pioneering studies of isotope-based characterisation of biological dehalogenation [12,13] is of high interest. Both Southern-blot and PCR-based approaches failed to detect the *dcmA* gene in strain MC8b [14]. Analysis of the ribosomal 16S rRNA gene indicated its affiliation to the Alphaproteobacterial genus *Hyphomicrobium*, but no further molecular analysis was attempted.

In this work, we used strain MC8b to demonstrate the value of a pan-proteomics-based approach circumventing whole-genome DNA sequence analysis for rapid strain interrogation of its key function of interest. Using a custom database of the predicted proteomes of available genome-sequenced *Hyphomicrobium* strains, global differential proteomics of DCM and methanol cultures showed that strain MC8b features a DCM utilisation system that is highly divergent from previously characterised DCM-degrading strains. This was confirmed by sequencing the genome of strain MC8b, which was obtained as a high quality, assembled, and closed sequence.

## 2. Materials and Methods

### 2.1. Strain Cultivation

Strain MC8b [12] was restreaked on solid mineral minimal medium [15] from a frozen laboratory stock, with DCM as the sole carbon and energy source for growth. For liquid cultures, the strain was routinely grown aerobically in the same medium, in gas-tight Erlenmeyer flasks fitted with Mininert caps (Supelco, Sigma, St. Louis, MO, USA), at 30 °C with agitation at 120 rpm, with either 10 mM DCM or methanol as carbon and energy source. Liquid cultures for proteomic analysis (25 mL; 5 biological replicates each with DCM or with methanol) were obtained as described above, starting from independent colonies of the strain grown with DCM on a solid medium. Upon reaching the late stationary phase, each preculture was added to a 1 L Erlenmeyer flask containing 200 mL mineral medium and 10 mM DCM or methanol and further cultivated as above. DCM-grown cultures were harvested during the exponential phase (OD_600_ 0.03–0.09) after 5 days, 130–200 mL aliquots centrifuged at 4 °C at 8000 rpm for 10 min, and cell pellets stored at −80 °C until further analysis. Methanol-grown cultures were also harvested in the exponential phase (OD_600_ 0.16–0.21), and 45 mL culture aliquots were processed in the same way as DCM cultures to yield similar amounts of cell material for the two conditions (Supplementary Table S1).

### 2.2. Cell Lysis and Enzymatic Proteolysis

Proteins were extracted from cell pellets as described previously [16]. Briefly, 1.7 mg of bacterial paste were resuspended in 100 μL of lithium dodecyl sulfate (LDS) 1X lysis buffer consisting of 106 mM Tris/HCl, 141 mM Tris base, 2% LDS (*w*/*v*), 10% glycerol (*w*/*v*), 0.51 mM EDTA, 0.22 mM SERVA Blue G-250, and 0.175 mM phenol red, buffered at pH 8.5 and supplemented with 5% beta-mercaptoethanol (*v*/*v*), boiled at 99 °C for 5 min, and then sonicated 5 min in an ultrasonic water bath (VWR ultrasonic cleaner). Resulting suspensions were transferred into 2 mL screw-cap microtubes (Sarstedt, Nümbretch, Germany) containing 200 mg of 0.1 mm silica beads (MP Biomedicals, Solon, OH, USA) and further lysed using a Precellys Evolution instrument (Bertin Technologies, Montigny-le-Bretonneux, France) operated with 3 cycles of 30 s at 7800 rpm and 30 s of pause between each cycle. After lysis, samples were centrifuged at 16,000× *g* for 1 min, and the supernatant was collected to a new microcentrifuge tube before incubation at 99 °C for 5 min. Enzymatic digestion was performed as previously described [17]. Proteins (15 µg, i.e., 25 µL at 0.6 µg/µL) were subjected to a short electrophoresis migration (5 min) on NuPAGE 4–12% Bis-Tris gel, at 200 V in MES/SDS 1X running buffer. The whole proteome from each sample was recovered by excision of a unique gel band. Proteins were then reduced using 25 mM dithiothreitol (Sigma-Aldrich, St. Louis, Missouri, USA) in 50 mM NH_4_HCO_3_ (Sigma-Aldrich) at 56 °C for 10 min and alkylated with 55 mM iodoacetamide (Sigma-Aldrich) in 50 mM NH_4_HCO_3_ for 10 min at room temperature in the dark. Proteins were proteolyzed with the addition of 0.4 μg of trypsin gold (Promega, Madison, WI, USA) in 0.01% of Protease Max surfactant (Promega) and 50 mM NH_4_HCO_3_ per sample. After 15 min incubation on ice, excess trypsin was removed, and 50 μL of 50 mM NH_4_HCO_3_ containing 0.01% of Protease Max surfactant were added. Proteolysis was performed at 50 °C for 60 min, followed by acidification of the resulting extracted peptides with trifluoroacetic acid (TFA) to 0.5% final concentration.

### 2.3. Mass Spectrometry and Data Interpretation

Liquid chromatography-tandem mass spectrometry (LC-MS/MS) analysis was performed using an Ultimate 3000 nano-LC system coupled to a Q-Exactive HF mass spectrometer (Thermo Scientific, Waltham, MA, USA) operated as described previously [18]. Peptide samples (2 µL, corresponding to approximately 200 ng of peptides) were loaded on a reverse-phase PepMap 100 C18 μ-precolumn (5 μm, 100 Å, 300 μm i.d. × 5 mm, Thermo Fisher, Waltham, MA, USA) and then resolved on a nanoscale PepMap 100 C18 nanoLC column (3 μm, 100 Å, 75 μm i.d. × 50 cm, Thermo Fisher) at a flow rate of 0.3 μL·min^−1^ using a 90 min gradient (4% B for 0 to 3 min, 4–25% B from 3 to 78 min, and 25–40% B from 78 to 93 min) of mobile phases A (0.1% HCOOH/100% H_2_O) and B (0.1% HCOOH/80% CH_3_CN/20%H_2_O). The mass spectrometer was operated in Top20 mode, with a scan range of MS acquisition from 350 to 1800 *m*/*z* and selection and fragmentation using 10 s dynamic exclusion time for the 20 most abundant precursor ions. Only ion precursors with a 2^+^ or 3^+^ charge were selected. HCD fragmentation was performed using a 27 eV normalized collision energy. Secondary ions were isolated with a window of 1.6 *m*/*z*.

The pan-proteomics database was built by merging the predicted proteomes from the annotated genomes of 13 *Hyphomicrobium* strains: *Hyphomicrobium* sp. NDB2Meth4, *Hyphomicrobium* sp. CS1GBMeth3, *Hyphomicrobium* sp. CS1BSMeth3, *Hyphomicrobium zavarzinii* ATCC 27496, *Hyphomicrobium* sp. 99, *Hyphomicrobium* sp. 802, *Hyphomicrobium nitrativorans* NL23, *Hyphomicrobium* sp. MC1, *Hyphomicrobium sulfonivorans*, *Hyphomicrobium facile*, *Hyphomicrobium denitrificans* 1NES1, and DCM-degrading strains *Hyphomicrobium denitrificans* ATCC 51888 and *Hyphomicrobium* sp. GJ21 (downloaded from NCBI in June 2018). The resulting database comprised 47,432 polypeptide sequences for a total of 14,834,376 residues. MS/MS spectra were assigned with this database using Mascot Daemon software version 2.6.1 (Matrix Science, London, UK), set with 5 ppm peptide tolerance and 0.02 Da MS/MS fragment tolerance, 2^+^ and 3^+^ peptide charge, a maximum of two missed cleavages, carbamidomethylation of cysteine as fixed modification, oxidation of methionine as variable modification, and trypsin as proteolytic enzyme. Peptides identified at *p*-value ≤ 0.05 in homology threshold mode and proteins identified with at least two distinct peptides were parsed using IRMa 1.31.1c software [19]. The false-positive rate was estimated to be below 1% for protein identification with the MASCOT decoy option search. Shared peptides were assigned with the most detected protein as assessed by specific peptides. Spectral counts, defined as the number of MS/MS spectra assigned per protein, were counted for all validated proteins using only non-ambiguous peptides as previously described [20]. Comparison of protein abundance between culture conditions was performed using the TFold test [21] and by defining four statistical groups: blue for a fold-change ≥ 1.5 and a *p*-value ≤ 0.05, orange for a *p*-value ≤ 0.05 and fold-change ≤ 1.5, green for a fold-change ≥ 1.5 and a *p*-value ≥ 0.05, and red for a fold-change ≤ 1.5 and a *p*-value ≥ 0.05. Data interpretation using the annotated genome of *Hyphomicrobium* sp. MC8b was done similarly, with proteins validated on the basis of at least two distinct peptide sequences. In this case, no parsimony rule was used, and spectral counts were evaluated by using only non-ambiguous peptides as previously described [20].

### 2.4. Genome Sequencing, Assembly and Annotation

Total DNA was prepared from a DCM-grown culture of strain MC8b using the MasterPure Complete DNA and RNA purification kit (Epicentre, Madison, WI, USA), flash-frozen and kept at −80 °C until further use. The DNA library for long-read sequencing was prepared using the Genomic DNA ligation kit (cat. no. SQK-LSK 108, Oxford Nanopore Technologies, Oxford, UK) according to the protocol provided, with a few modifications. Briefly, the DNA repair and end preparation steps were merged, and the DNA purification step between end preparation and native barcode ligation was omitted. After adaptor ligation, the library was purified using 0.6X AMPure XP beads (cat. no. A63881, Beckman Coulter, Brea, CA, USA). The resulting library was loaded on the flowcell (FLO-MN106) and sequenced on a MinION (Oxford Nanopore Technologies). After 24 h, the freshly prepared adaptor-ligated library was loaded again on the same flowcell. For short-read sequencing, 200 ng DNA was processed for library preparation using the KAPA HyperPlus kit (cat.no. 07962410001, Roche, Basel, Switzerland) without PCR amplification, according to the manufacturer’s protocol. Enzymatic fragmentation time was 20 min, aiming at 300 bp average fragment size for the 2 × 150 bp paired-end sequencing run. The library was quantified using Qubit (Invitrogen, Carlsbad, CA, USA), its quality assessed by Bioanalyzer (Agilent, Santa Clara, CA, USA), and sequenced using NextSeq500 (Illumina, San Diego, CA, USA) at the Luxembourg Center for Systems Biomedicine (LCSB) sequencing platform (University of Luxembourg).

Raw ONT sequence data were base-called using Albacore Sequencing Pipeline Software (version 2.3.3) with r94_450bps_linear.cfg to match flowcell FLO-MIN106 and kit SQK-LSK108. Base-called reads were size-selected to be at least 1 kbp in length. Illumina sequence data were preprocessed by fastp (version 0.19.5) [22], and only properly paired, preprocessed reads were conserved.

Unicycler (version 0.4.7) [23] was used to assemble the genome from Illumina and Nanopore reads with the following options: --threads 28, --no_rotate -1, FORWARD_ILLUMINA_READS.fq.gz -2, REVERSE_ILLUMINA_READS.fq.gz –l, and SIZE_SELECTED_ONT_READS.fq. Associated softwares were SPAdes (version 3.13.0), racon (version 1.3.1), bowtie2-build (version 2.3.4.3), bowtie2 (version 2.3.4.3) [24], samtools (version 1.9) [25], java (version 1.8.0_162), and pilon (version 1.23) [26]. CheckM (version 1.0.12) [27] was used to verify assembly completeness with the following parameters: lineage_wf -t 12 -f CHECKM_RESULTS_FILE.txt -x fasta DIRECTORY_OF_UNICYCLER_ASSEMBLY OUT_DIRECTORY. The dependencies of CheckM and their respective versions were HMMER (version 3.2.1) [28], prodigal (version 2.6.3) [29], pplacer (version 1.1.alpha17) [30], and Python (version 2.7.14).

### 2.5. Data

Mass spectrometry proteomics data were submitted to the ProteomeXchange Consortium via the PRIDE partner repository under dataset identifiers PXD021816 and 10.6019/PXD021816 for the pan-proteomics study and PXD021817 and 10.6019/PXD021817 for the MC8b-genome derived proteomics analysis. The assembled genome was automatically annotated at Genoscope using the MicroScope pipeline [31] and uploaded on the MicroScope web platform (https://mage.genoscope.cns.fr/microscope) for genome sequence analysis, and the obtained annotations were used for proteomics analysis. The genome sequence data for this study were deposited in the European Nucleotide Archive (ENA) at EMBL-EBI under accession number PRJEB40967 (https://www.ebi.ac.uk/ena/browser/view/PRJEB40967).

## 3. Results

Preliminary experiments confirmed that DCM-degrading strain MC8b was also capable of growing with methanol, the reference growth substrate for strains of the *Hyphomicrobium* genus [32] to which strain MC8b had been tentatively affiliated [14]. We hypothesised that synthesis of the required dehalogenase to sustain strain MC8b growth on DCM may be regulated by the presence of its growth substrate, as observed in many dehalogenating strains [33]. Thus, we performed rapid differential proteomic analysis by pan-proteomics of cultures of the strain grown with either DCM or methanol.

### 3.1. Pan-Proteomics Strategy for Characterizing DCM-Degrading Strain MC8b of Unknown Genome Sequence

**Figure 1:**
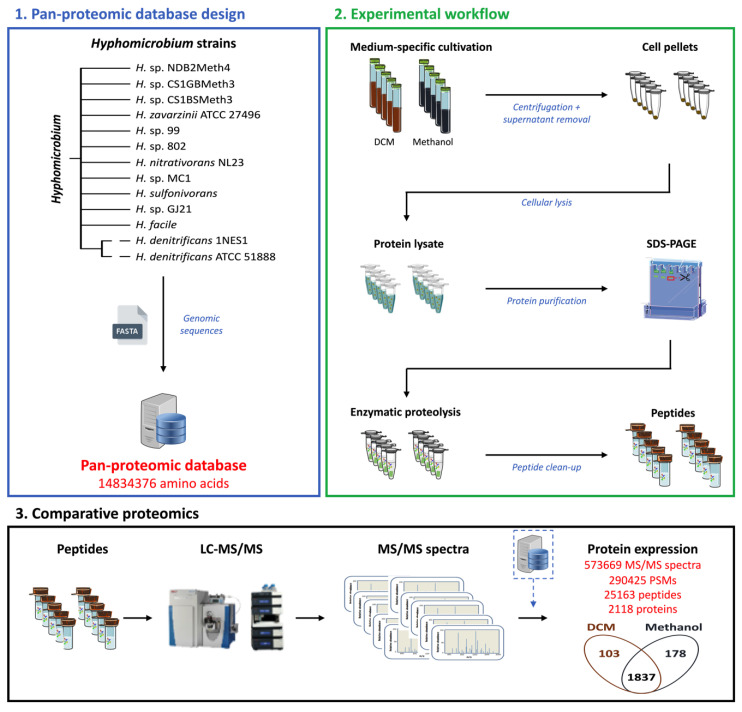
Pan-proteomics workflow to decipher key protein determinants of the dichloromethane utilisation pathway from the *Hyphomicrobium* sp. MC8b strain. DCM: dichloromethane.

The pan-proteomics approach used to rapidly characterise the proteome of *Hyphomicrobium* sp. MC8b strain of unknown genome sequence (Figure 1) consisted of using a database with the predicted proteomes of 13 taxonomically closely related strains to *Hyphomicrobium* sp. MC8b (listed in Materials and Methods). Strain MC8b was grown with DCM or with methanol as the sole carbon and energy source, in quintuple replicates for each condition (Supplementary Table S1), and comparative proteomics were performed by nanoLC-MS/MS and interpretation of the recorded spectra using the pan-proteomics database.

### 3.2. Global Changes in the Proteome Hyphomicrobium sp. MC8b upon Growth with Dichloromethane

**Figure 2:**
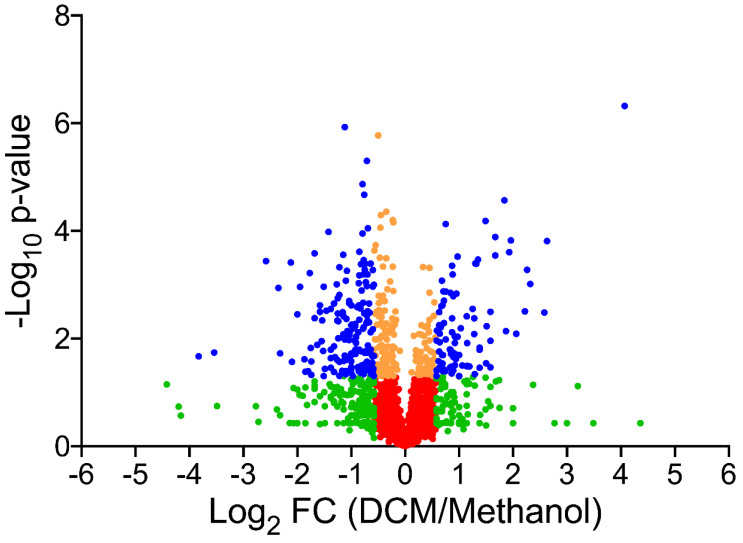
Comparative pan-proteomics analysis of *Hyphomicrobium* sp. MC8b. Volcano plot of protein abundances under dichloromethane versus methanol growth conditions. Proteins are distributed depending on their abundance fold-change and *p*-value. Blue proteins show significant fold-change (≥1.5) and are significantly differentially abundant (*p* value ≤ 0.05). Proteins in orange (*p*-value ≤ 0.05 but fold-change ≤ 1.5), green (fold-change ≥ 1.5 but *p*-value ≥ 0.05), and red (fold-change ≤ 1.5, *p*-value ≥ 0.05) were not further considered.

In total, 573,669 spectra were recorded from quintuple replicates of strain MC8b grown with DCM or with methanol and mapped against the pan-proteomics database. We assigned 290,425 spectra to peptide sequences and detected 2118 proteins in total (Supplementary Table S2), confirming that strain MC8b is affiliated to *Hyphomicrobium* (4329 CDS on average in the sequenced *Hyphomicrobium* strains of the pan-proteomics database). Of the identified proteins, 281 showed differential abundance between DCM and methanol conditions as assessed by label-free shotgun proteomics, with 103 proteins more abundant with DCM (Figure 1 and Figure 2 and Supplementary Table S3), indicating that growth with DCM requires significant molecular adjustments. Differentially abundant proteins present in the two DCM-degrading strains in the pan-proteomics database, *Hyphomicrobium* strains *H. denitrificans* ATCC 51888 [34] and *H.* sp. GJ21 [35], were not over-represented (Supplementary Figure S1), suggesting that bacterial adaptation to DCM utilisation strongly involves the core genome of *Hyphomicrobium*.

### 3.3. Proteomics-Driven Identification and Sequence Prediction of Strain MC8b DCM Dehalogenase

Four abundant peptide sequences specifically matching DcmA DCM dehalogenase of known genome sequence were unexpectedly obtained, although PCR amplification of the strongly conserved *dcmA* gene had been unsuccessful [14]. DCM dehalogenase DcmA was actually the protein with the highest differential abundance between DCM and methanol conditions (Supplementary Table S3). However, these four peptides accounted for only 18% coverage of the full-length protein. This suggested that strain MC8b features a divergent DcmA sequence compared to the very conserved sequence of all experimentally characterised Alphaproteobacterial DCM-degrading strains. We explored this hypothesis by analysing obtained tandem mass spectra using a custom set of_DcmA proteins of *Methylobacterium extorquens* DM4, *H.* sp. GJ21, *H. denitrificans,* and *Methylophilus* sp. DM11 strains in error-tolerant mode, in order to access peptide sequences differing slightly from known DcmA sequences. In this way, eight additional peptide sequences were defined from all five replicate cultures grown with DCM, yielding additional 40% coverage of a full-length DCM dehalogenase protein (Table 1). The predicted DcmA sequence of strain MC8b strongly differed from the 96–99% identical DCM dehalogenases of Alphaproteobacterial DCM-degraders [36]. Out of the total 924 spectral counts assigned to DcmA in error-tolerant mode, only 12 were observed in the methanol condition and for two of the total twelve detected peptides only. This further indicated that the synthesis of this protein, like that of all characterised DcmA homologs, is highly dependent on the presence of DCM.

In all DCM-degrading strains with DcmA investigated so far, the gene cluster encoding DcmA also featured near-identical genes *dcmR*, which is involved in the regulation of DCM dehalogenase gene expression, and *dcmB* and *dcmC* of unknown function [11]. At only 30% sequence identity at the protein level, the closest functionally annotated protein to DcmB is a cephalosporin biosynthesis protein involved in hydroxylation/methyl transfer [37]. DcmC lacks functionally annotated full-length relatives, an unusual cysteine protease [38] with 29% identity over 50 amino acids being its closest annotated match. The corresponding gene products DcmR, DcmB, and DcmC were all detected in a recent proteomics study of *M. extorquens* DM4 and also showed higher abundance with DCM [39]. Here, DcmR and DcmB were also detected (Supplementary Table S3), but again with poor coverage. In contrast, DcmC was not detected, unlike its homolog in strain DM4 [39].

### 3.4. The Genome of Strain MC8b Features the Most Divergent Set of Dcm Genes Known So Far

Insights about the atypical DCM dehalogenation system of strain MC8b obtained by rapid genome-independent pan-proteomics analysis warranted sequencing of its genome. Sequencing of total DNA from DCM-grown cultures of strain MC8b using a combination of Oxford Nanopore and Illumina technologies yielded a circularised high-quality sequence for this strain. No plasmids were detected. Genome analysis was performed on the MicroScope platform at Genoscope [31]. At 4274 kb in size, the genome sequence of strain MC8b is typical of the *Hyphomicrobium* genus, with a single rRNA operon and a GC content of 59.65%. The genome was predicted to be complete by checkM [27] (100% completeness and 0.2% contamination with 1 marker duplicated). In total, 4574 CDS were predicted from the genome sequence [31]. Comparative analysis with other *Hyphomicrobium* genomes showed that strain MC8b is most closely related to the chloromethane-degrading strain *Hyphomicrobium* sp. MC1 [40] and to *H.* sp. 802, with about 80% closely homologous protein-encoding genes in synteny between the genome of strain MC8b and these two genomes.

We then checked whether the protein sequence of strain MC8b DCM dehalogenase predicted by pan-proteomics was confirmed by genome sequencing. In addition, we investigated how efficient analysis of our ad hoc pan-proteomics database of 13 predicted proteomes from genome-sequenced *Hyphomicrobium* strains had been in defining the DCM-specific proteome of strain MC8b, comparing it to an analysis performed with the predicted proteome of strain MC8b derived from its genome sequence.

**Figure 3:**
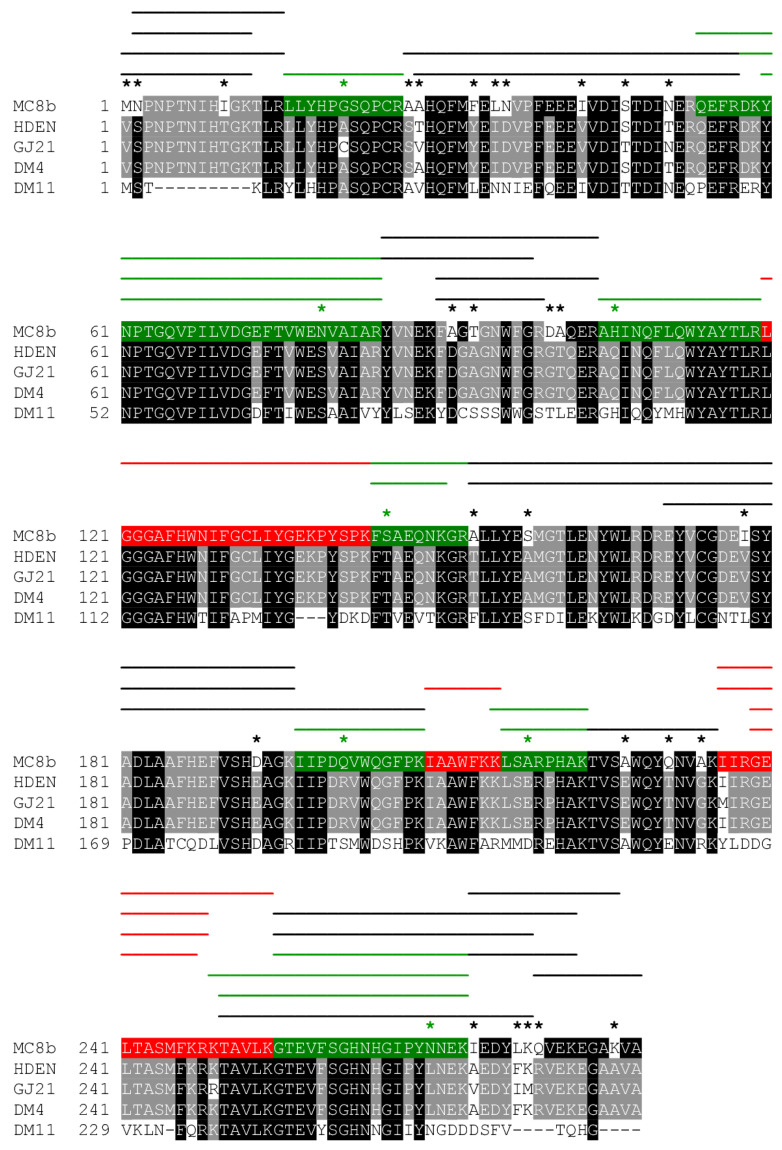
Sequence alignment of selected DCM dehalogenases covering the known sequence diversity of DCM dehalogenases, highlighting the highly divergent sequence of *Hyphomicrobium* sp. MC8b DcmA sequence detected and partially predicted using pan-proteomics analysis, and verified by sequencing of the MC8b genome. All peptides detected using the pan-proteomics *Hypomicrobium* database analysed in normal or error-tolerant mode are shown in red and green, respectively. Additional peptides detected by analysis of obtained spectra using the predicted proteome of strain MC8b based on the genome sequence of the strain are shown by black lines. A star denotes a sequence difference in the DcmA sequence of strain MC8b compared to that of strain *H.* sp. GJ21 and/or *H. denitrificans* ATCC 51888 (HDEN). Green stars indicate sequence variations in the MC8b DcmA sequence that prevented detection of peptides by analysis of the *Hyphomicrobium* database in the absence of error tolerance, and black stars indicate sequence variations extending beyond the one-mismatch threshold for peptide detection in error-tolerant mode, respectively.

The eight DcmA peptides predicted by pan-proteomics analysis in error-tolerant mode, together with the four peptides identical to known DcmA sequences (Table 1), were validated by the genome sequence of strain MC8b, confirming that its DCM dehalogenase significantly differed from other known Alphaproteobacterial sequences, with only the sequence of Betaproteobacterium *Methylophilus* sp. strain DM11 being more divergent [36] (Figure 3). Interestingly, the peptide correctly identified as YVNEKFAGTGNWFGR in the genome-based proteomic analysis had been predicted as the isomeric sequence YVNEKFTGAGNWFGR in the original analysis with the pan-proteomics database (Table 1). This is because the peptide sequence derived from the DNA sequence involves a two-residue difference from sequences in the pan-proteomics database, above the single residue mismatch threshold of error-tolerant proteomic analysis that is computationally practicable today.

**Figure 4:**
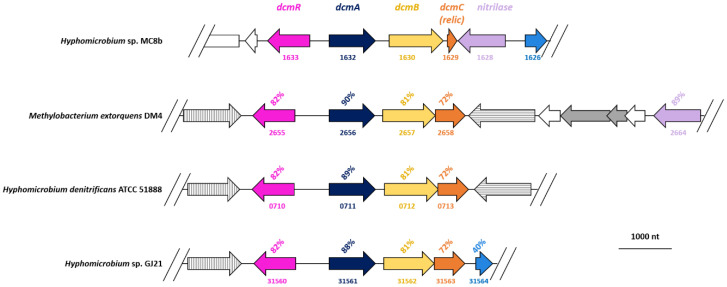
Comparison of *dcm* genes of *Hyphomicrobium* strains MC8b, ATCC 51888, and GJ21, and of the reference DCM-degrading Alphaproteobacterial strain *Methylobacterium extorquens* DM4. Homologous genes are shown in the same colour, with percentage identities to strain MC8b given at the protein level. Transposase genes are shown in grey, with homologous genes showing the same fill pattern. Numbers under the arrows for *Hyphomicrobium* strains ATCC 51888 and GJ21 and *Methylobacterium extorquens* strain DM4 refer to corresponding gene identifiers in Genbank.

Analysis of the genome context of gene *dcmA* gave further indications that the *dcmA*-dependent DCM dehalogenation system of strain MC8b differed from that encoded by the strongly conserved *dcmRABC* gene cluster of other DCM-degrading Alphaproteobacteria (Figure 4). DcmA, DcmB, and DcmR differ significantly from their homologs in other DCM-degrading strains. Further, gene *dcmC* was only present as a relic in strain MC8b (Figure 4). The corresponding short (57 aa) orf downstream of DcmB showed only 68% identity over 17 residues with the 184-residue reference DcmC of *Hyphomicrobium* strains ATCC 51888 and GJ21 and of strain DM4, and remained undetected by proteomics. The strong sequence variation observed in strain MC8b in the genomic context of *dcmA*, including the absence of transposases, usually flanking *dcm* genes in DCM-degrading strains (Figure 4), also explained the failure to detect the *dcm* gene cluster until now and confirmed that strain MC8b features the most divergent DcmA-based system discovered so far.

### 3.5. Further Insights from Proteomic Analysis Underlines the Power of the Pan-Proteomics Approach

**Figure 5:**
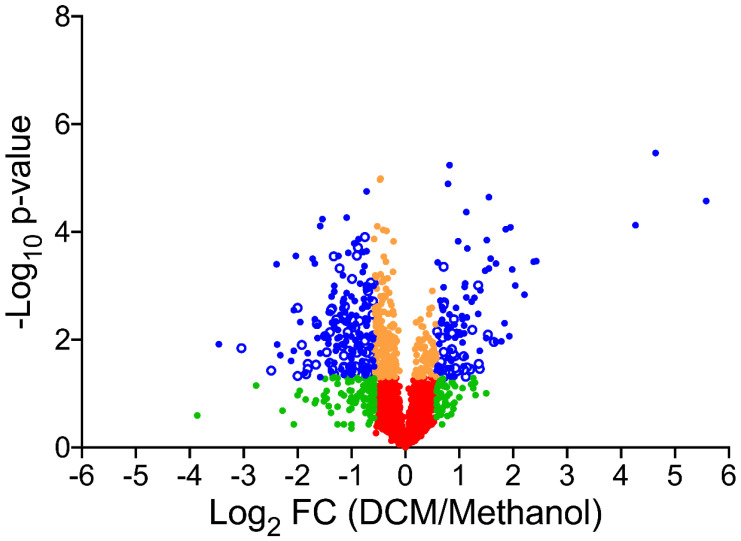
*Hyphomicrobium* sp. MC8b differential proteomics using the genome sequence of the strain. Volcano plot of protein abundances in dichloromethane versus methanol growth conditions. Colour code is as in Figure 2, with open blue symbols highlighting proteins with significant differential abundance that were additionally identified from the proteome of strain MC8b predicted from the genome sequence.

In order to assess the value of results previously obtained by the pan-proteomics approach without a genome sequence for strain MC8b, proteomic data were then matched to the predicted proteome of strain MC8b derived from its genome sequence. In comparison to the 2118 proteins identified using the pan-proteomics database, 2101 were confirmed with the predicted proteome of strain MC8b. With regard to differentially abundant proteins (Figure 5), 328 proteins were identified with the specific MC8b proteome database (Supplementary Table S4), as compared to 281 with the *Hyphomicrobium* pan-proteomics database (Supplementary Table S3). About a third (126) additional proteins were identified as more abundant with DCM using the strain-specific proteome (Figure 5, Supplementary Table S4). Along the same lines, we also checked whether the distribution of COG categories [41] for proteins with significant differential abundance between DCM and methanol conditions differed from that encoded in the genome. Proteins associated with DNA repair (L) and cell wall structure and biogenesis (M) were over-represented in the proteome (Supplementary Table S5). Conversely, proteins associated with transcription and translation were under-represented. This was not only observed in the analysis of the MC8b proteome derived from its genome sequence but already in the initial analysis with the ad hoc database constructed from the theoretical proteomes of the 13 *Hyphomicrobium* strains of known genome sequence (Supplementary Table S5). This confirmed the power of the pan-proteomics approach for rapid analysis of strains with functions of interest, within a well-investigated taxonomical framework, and in the absence of specific genome sequence information.

## 4. Discussion

The pan-proteomics concept [4,7] can be applied to a relatively large number of prokaryotic isolates, as the genomes of many types of microorganisms have now been sequenced. While the small number of available genomes for eukaryotes still represents a limitation for the application of pan-proteomics, the similar concept of “homology-driven proteomics” [42,43] has proved helpful for the analysis of proteomes of unsequenced animals and plants [44,45]. The power of the pan-proteomics approach was supported by the results obtained in the present study. First, it was unexpected that strain *Hyphomicrobium* sp. MC8b contains DCM dehalogenase DcmA. The atypical DcmA-based system of strain MC8b was evidenced by unprecedented differences in DcmA and DcmB sequences, while DcmC remained undetected. Pan-proteomics results were then confirmed by genome sequencing. Notably, the gene encoding the differentially abundant putative nitrilase (Figure 4, Table S3), whose homolog is located nearby *dcmRABC* genes in the genome of *M. extorquens* DM4 (Figure 4), was found next to the *dcmC* relic in the MC8b genome, suggesting that it may be associated with DCM metabolism as well.

Indeed, the possibility of analysing differential abundance in the condition of interest compared to a reference condition represents a strong asset to identify proteins involved in a function of interest by pan-proteomics. In the field of bacterial dehalogenation, in particular, synthesis of the proteins involved in dehalogenation of a given organohalide often depends on its presence [33]. Identification of proteins of interest for a particular function will be more challenging when only one cultivation condition is available. For *Candidatus* Dichloromethanomonas elyunquensis growing with DCM under strictly anoxic conditions, for example, potential dehalogenase candidates were tentatively proposed based on DCM-grown cultures as the only available growth condition for this system [46].

Differential pan-proteomics may also provide clues on associated key proteins and potentially also corresponding metabolic pathways and adaptations related to the function of interest. For instance, proteomic analysis of the reference DCM-degrading strain *M. extorquens* DM4 [39] suggested that DCM metabolism by a DcmA-dependent system triggers adaptations related to DNA genotoxicity, acid and chloride production, and membrane integrity, thereby confirming previous work based on mutagenesis studies [11,47]. These findings were confirmed here at the level of general gene functional classes (COGs) (Supplementary Table S5). The observed shift in COG distribution of proteins more abundant in the DCM condition compared to that in the theoretical proteome predicted from the genome sequence highlighted general functions associated with DCM metabolism. This shift was already detected by pan-proteomics analysis, i.e., without knowledge of the strain-specific theoretical proteome (Supplementary Table S5).

Nevertheless, the modest overlap in proteins with differential abundance with the previous study on the reference DCM-degrading strain *M. extorquens* DM4 is noteworthy. Only nine proteins, including DCM dehalogenase DcmA and DcmB protein of unknown function, were identified as differentially abundant in both *Hyphomicrobium* strain MC8b and *M. extorquens* strain DM4 (Supplementary Table S3). On the one hand, two of the proteins detected as differentially abundant in both DM4 and MC8b proteomics studies had already been associated with DCM metabolism. The squalene hopene cyclase *shc* gene was identified as essential for growth with DCM [47,48], while a transglycosylase/transpeptidase homolog (HYPMC8B_3734, annotated as penicillin-binding protein; METDI4661 in strain DM4) showed DCM-dependent synthesis [47]. These findings confirm the likely importance of these two proteins as part of a specific ensemble of proteins associated with bacterial growth on DCM and involving DNA repair and envelope processes (Supplementary Table S5). On the other hand, the small number of differentially abundant proteins shared by strains MC8b and DM4 growing with DCM contrasts with the fact that the two strains share 1136 homologous proteins with over 50% identity at the protein level, with a similar number of detected proteins in the two studies (2453 proteins for strain MC8b versus 2878 for strain DM4 [39]). To us, this suggests that adaptation to dehalogenation of DCM involves specific changes in expression of the taxonomically defined core genome following acquisition of genes for DCM utilisation [49,50], in keeping with the broad functional categories associated with transformation of DCM (Supplementary Table S5), and as suggested by transcriptional studies [51]. In other words, genes involved in adaptation to DCM are not limited to a specific set of genes of DCM-degrading strains, as evidenced by differentially abundant proteins shared by many and sometimes all 13 *Hyphomicrobium* strains of the pan-proteomics database (Supplementary Figure S1).

Finally, and with regard to the process of horizontal transfer of *dcm* genes itself, the lack of IS elements flanking *dcm* genes in strain MC8b, as well as the unusual arrangement and sequence of its *dcm* gene cluster (Figure 4), raises new questions on the evolution and subsequent dissemination of the capacity to grow with DCM in the bacterial world. Clearly, the *dcm* gene cluster of DCM-degrading strains with DcmA DCM dehalogenase may not be as conserved or as essential in defining DCM-degrading strains as generally believed until now.

## 5. Conclusions

Pan-proteomics allowed us to uncover a hitherto undetected dehalogenase enzymatic system in strain MC8b. Moreover, differential proteomics allowed to identify DCM-induced proteins that may also be associated with dehalogenation. Thus, a custom pan-proteomics database allows one to propose proteins involved in key functions of interest in the absence of genome sequence. The power of this approach will increase with the number of sequenced genomes in databases, and make possible fast fingerprinting of strain metabolism under conditions where genome or transcriptome sequencing is unfeasible.

The results obtained for strain MC8b also put the evolution of DCM dehalogenases in a new light. Indeed, the strong differences in *dcm* genes observed for strain MC8b strongly suggest that horizontal gene transfer, while important today in environments contaminated with DCM, may represent a relatively recent feature of the evolution of DCM dehalogenases capable of supporting bacterial growth with DCM. This, to us, is an incentive to renew explorations of enzymatic systems of DCM dehalogenation using state-of-the-art function-based approaches. More generally, pan-proteomics may help to rapidly discover in unsequenced strains still unchartered solutions developed by the microbial world to degrade organohalides over the eons.

## Figures and Tables

**Table 1:** Peptide sequences for DcmA of strain MC8b predicted by proteomic analysis.

First	Last	Sequence ^1^	#Peptides ^2^	Analysis
16	26	LLYHPGSQPCR	1	error-tolerant mode
54	84	QEFRDKYNPTGQVPILVDGEFTVWENVAIAR	3	error-tolerant mode
85	99	YVNEKFTGAGNWFGR	2	error-tolerant mode
105	119	AHINQFLQWYAYTLR	1	error-tolerant mode
120	143	LGGGAFHWNIFGCLIYGEKPYSPK	1	no mismatch
144	152	FSAEQNKGR	2	error-tolerant mode
153	168	ALLYEAMGTLENYWLR	3	error-tolerant mode
197	208	IIPDQVWQGFPK	2	error-tolerant mode
209	215	IAAWFKK	1	no mismatch
215	223	KLSARPHAK	2	error-tolerant mode
236	248	IIRGELTASMFKR	4	no mismatch
248	254	RKTAVLK	1	no mismatch

^1^ Residues in green highlight amino acid variations from reference DcmA sequences detected in error-tolerant mode. ^2^ Number (#) of different peptides detected by LC-MS/MS that correspond to all or part of the indicated sequence.

## Supplementary Materials

The following are available online at https://www.mdpi.com/2076-2607/8/12/1876/s1. Table S1: Cultures used for comparative proteomics analysis; Table S2: Overview of obtained data from LC MS/MS analysis of the proteome of strain MC8b grown with DCM or methanol; Table S3: Proteins detected by pan-proteomics. See separate Excel file; Table S4: Proteins detected from the annotated genome sequence of *Hyphomicrobium* sp. MC8b. See separate Excel file; Table S5: Distribution (in percent) of COG categories for differentially abundant proteins of *Hyphomicrobium* sp. MC8b analysed with the pan-proteomics database or the MC8bgenome-derived database, and for the total predicted proteome of strain MC8b; Figure S1: Proteomes in the pan-proteomics database.

## Abbreviations

CDS	coding DNA sequence
COG	cluster of orthologous groups
DCM	dichloromethane
LC-MS/MS	liquid chromatography-tandem mass spectrometry
PSM	peptide spectrum match
