# Supplementary material for: Dichloromethane Degradation Pathway from Unsequenced Hyphomicrobium sp. MC8b Rapidly Explored by Pan-Proteomics
Source: Microorganisms. 2020 Nov 27;8(12):1876. doi: 10.3390/microorganisms8121876 (PMC7760279; doi:10.3390/microorganisms8121876)
Supplement: Supplementary file 1 [file microorganisms-08-01876-s001.zip › microorganisms-1016562-sl proof/Hayoun_SupplementaryTables_S1_S2_S5.pdf]

Supplementary materials

Supplementary Table S1. Cultures used for comparative proteomics analysis.

Replicate	Growth substrate	Final OD_{600}	Cell pellet (mg wet weight)	Culture volume (ml)
1	DCM	0.07	16.7	200
2	DCM	0.09	17.6	130
3	DCM	0.03	13.2	200
4	DCM	0.06	15.7	180
5	DCM	0.07	10.2	200
6	Methanol	0.19	19.2	45
7	Methanol	0.19	17.7	45
8	Methanol	0.21	19.4	45
9	Methanol	0.20	19.4	45
10	Methanol	0.16	16.0	45

Supplementary Table S2. Overview of obtained data from LC MS/MS analysis of the proteome of strain MC8b grown with DCM or methanol.

Feature	Pan-proteomics [1]	MC8b [2]
Proteins in the database	21119	4570
Proteins detected	3394	2453
Proteins with at least 2 peptides	2118	2101
Total spectral counts (PSM) [3]	290425	345647
Total spectral counts, DCM condition	140083	167780
Total spectral counts, methanol condition	150342	177867
Differentially abundant proteins (fold-change >1.5, $p < 0.05$)	281	328
among which showing higher abundance with DCM	103	115
among which showing higher abundance with methanol	178	213
Proteins with fold-change >1.5 but $p > 0.05$	281	173
Proteins with fold-change <1.5 but $p < 0.05$	167	219
Proteins with fold-change <1.5 but $p > 0.05$	1389	1379

[1] Analysis with a database of predicted proteomes from 13 *Hyphomicrobium* strains of known genome sequence. [2] Analysis using the proteome of strain MC8b predicted from automatic annotation of its genome sequence using the MicroScope pipeline. [3] Total spectral counts for all 5 replicates of both DCM and methanol growth conditions.

Supplementary Table S3 . Proteins detected by pan-proteomics. See separate Excel file.

Supplementary Table S4. Proteins detected from the annotated genome sequence of *Hyphomicrobium* sp. MC8b. See separate Excel file.

Supplementary Table S5. Distribution (in percent) of COG categories for differentially abundant proteins of *Hyphomicrobium* sp. MC8b analysed with the pan-proteomics database or the MC8b genome-derived database, and for the total predicted proteome of strain MC8b.

Category		Differentially abundant proteins		Predicted proteome
		Pan-proteomics	MC8b	MC8b
D	Cell cycle control, cell division, chromosome partitioning	1.8	2.4	1.0
M	Cell wall/membrane/envelope biogenesis	10.4	10.7	5.7
N	Cell motility	1.4	1.8	1.2
O	Posttranslational modification, protein turnover, chaperones	3.6	3.0	3.4
T	Signal transduction mechanisms	4.7	4.3	4.4
U	Intracellular trafficking, secretion, and vesicular transport	1.1	0.9	1.7
V	Defense mechanisms	2.5	2.4	1.5
W	Extracellular structures	0.0	0.0	0.1
Z	Cytoskeleton	0.0	0.0	0.0
B	Chromatin structure and dynamics	0.0	0.0	0.0
J	Translation, ribosomal structure and biogenesis	1.4	1.8	3.8
K	Transcription	2.5	2.7	5.2
L	Replication, recombination and repair	6.8	7.3	3.1
C	Energy production and conversion	4.7	3.4	5.1
E	Amino acid transport and metabolism	6.1	7.0	6.8
F	Nucleotide transport and metabolism	0.4	0.3	1.3
G	Carbohydrate transport and metabolism	2.5	4.3	3.7
H	Coenzyme transport and metabolism	2.2	1.2	3.1
I	Lipid transport and metabolism	3.6	3.0	3.1
P	Inorganic ion transport and metabolism	4.3	3.7	4.9
Q	Secondary metabolites biosynthesis, transport and catabolism	1.8	1.5	2.4
R	General function prediction only	12.2	11.3	11.0
S	Function unknown	8.6	8.8	7.2
	Total proteins considered	278	328	4574
	Percentage of proteins with COG annotation	82.7	82.0	66.5
