# Supplementary figures and images for: Dichloromethane Degradation Pathway from Unsequenced Hyphomicrobium sp. MC8b Rapidly Explored by Pan-Proteomics
Source: Microorganisms. 2020 Nov 27;8(12):1876. doi: 10.3390/microorganisms8121876 (PMC7760279; doi:10.3390/microorganisms8121876)

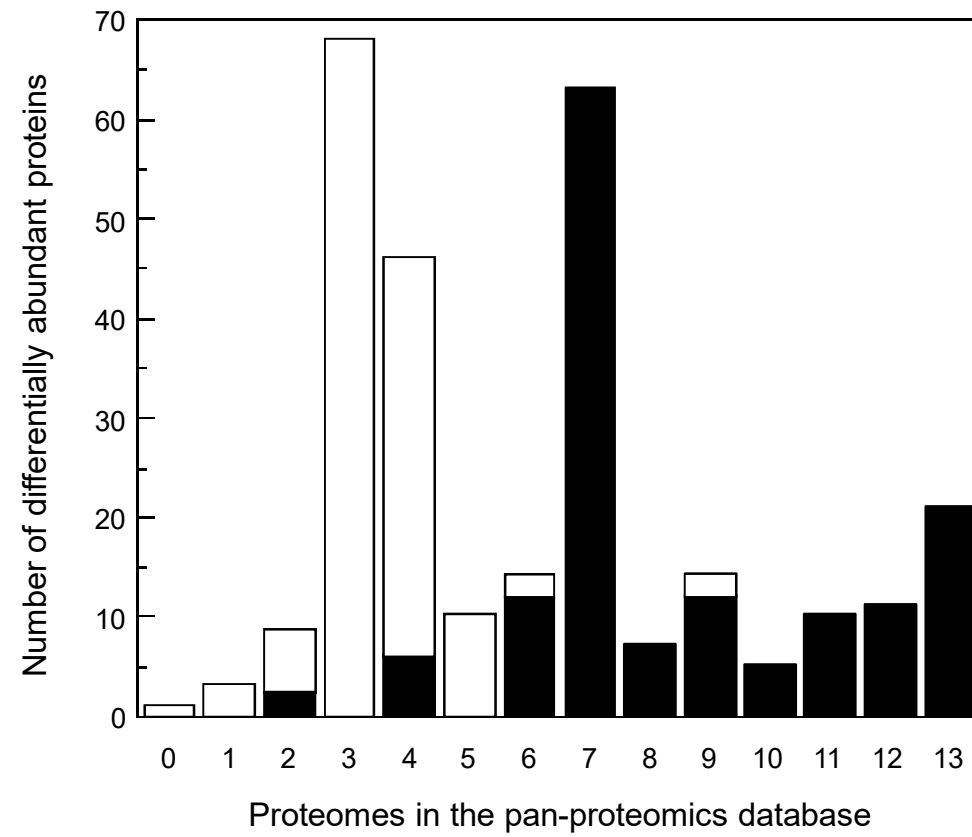

Supplement: Supplementary file 1 [file microorganisms-08-01876-s001.zip › microorganisms-1016562-sl proof/MC8b_FigS1.pdf]
